# Clinicians' Perspectives and Experiences of Barriers, Enablers and Implementation of a Child and Adolescent Mental Health Crisis Service: A Qualitative Evaluation of the Safeguards Team Program

**DOI:** 10.1111/jcap.70075

**Published:** 2026-09-09

**Authors:** Md Nazmul Huda, Teresa Winata, Weng Tong Wu, Thomas Nguyen, Michael Bowden, Valsamma Eapen, Rajeev Jairam

**Affiliations:** ^1^ Discipline of Psychiatry and Mental Health, School of Clinical Medicine, Faculty of Medicine and Health University of New South Wales Sydney New South Wales Australia; ^2^ Academic Unit of Infant, Child and Adolescent Psychiatry Ingham Institute for Applied Medical Research Liverpool New South Wales Australia; ^3^ Infant Child and Adolescent Mental Health Service, Mental Health South Western Sydney Local Health District Campbelltown New South Wale Australia; ^4^ National Disability Insurance Scheme Quality and Safeguards Commission Parramatta New South Wales Australia; ^5^ School of Medicine Western Sydney University Campbelltown New South Wales Australia; ^6^ Sydney Medical School University of Sydney Sydney New South Wales Australia; ^7^ Mental Health Branch, NSW Health Sydney New South Wales Australia; ^8^ Sydney Children's Hospitals Network Sydney New South Wales Australia

**Keywords:** barriers, facilitators, mental health crisis, service improvement, young people

## Abstract

**Background:**

Presentations of children and young people (CYP) to Emergency Departments (ED) for acute mental health (MH) issues have increased globally and in Australia. The Safeguards Teams Program (STP) was implemented to provide acute, rapid response, recovery‐focused, trauma‐informed brief intervention. This study aims to explore clinicians' perspectives and experiences implementing the STP, including barriers, facilitators and suggestions for programme improvement.

**Methods and Materials:**

This study used a qualitative approach by employing in‐depth interviews. Participants were recruited using purposive sampling. The interview transcripts were coded and analysed thematically.

**Results:**

Key themes included perceived barriers to implementation, programme enablers and suggestions for improvement. Enablers of successful implementation included structures that supported clinicians' outreach and service accessibility, a centralised and flexible referral model that clinicians perceived facilitated timely care, and opportunities for clinicians' capacity building and collaboration. Clinicians also perceived that the STP supported children and young people experiencing MH crises who may not meet the intake criteria for other services, thereby addressing an important service gap. Perceived barriers included high demand, which increased workload, and challenges associated with the model of care (MoC) in its early stages of implementation. Suggestions for programme improvement emphasised the need for greater contextualisation of services through co‐design, a more flexible MoC as part of STP service delivery, and a structured and formalised referral process. Participants also recommended including clinicians with specific skills and cultural awareness, enhancing community awareness, and strengthening integration efforts.

**Conclusions:**

Clinicians perceived the STP as a valuable crisis‐response service with the potential to address an important service gap for CYP who need rapid outreach specialist MH crisis response. Findings relating to clinicians' experiences and perceptions of implementing the STP may inform the development, refinement and implementation of similar crisis‐response models. Further quantitative research is needed to evaluate clinical outcomes, service utilisation and consumer perspectives associated with the STP.

## 1 Introduction

Over the past decade, there has been a global rise in the demand for specialised mental health services for children and young people (CYP), accounting for nearly half of the burden of disease in this age group, and this increase has far outpaced that observed in other age groups (Solmi et al. 2022). In Australia, several key indicators, such as psychological distress, deliberate self‐harm and suicide rates among young individuals, have shown a consistent upward trend, surpassing the rate of population growth (Australian Institute of Health and Welfare 2024). Notably, the steepest increases are observed among younger adolescents, whereby from 2008–2009 to 2021–2022, there was a greater than threefold increase in the rate of intentional self‐harm hospitalisations among those aged 14 and below (from 19 hospitalisations per 100,000 population to 72) (Australian Institute of Health and Welfare 2024). Such a surge in demand has been further worsened by the impact of the COVID‐19 pandemic (Sara et al. 2023). In such situations, clinicians in mental health crisis services have played a pivotal role in improving CYP's mental health (Nicholas et al. 2021). In the current study, clinicians refer to multidisciplinary mental health professionals, including psychiatrists, mental health nurses and social workers involved in the delivery of crisis mental health care.

Existing evidence indicates that child and adolescent mental health crisis services face several implementation and service‐delivery challenges. These include fragmented care pathways, difficulties coordinating services across hospital and community settings, workforce recruitment and retention problems, changes in senior management, weak communication between organisational leaders and the workforce, and inadequate resources and training (Kusnierczak et al. 2025; Paton et al. 2021; Reed et al. 2025). These challenges can reduce service accessibility, delay assessment and intervention, and make it difficult for clinicians to provide coordinated and sustainable crisis care. Although studies have identified barriers and facilitators affecting youth mental health service implementation, less is known about how these factors operate during the implementation of rapid, mobile, community‐based crisis response services for CYP.

In Australia, Child and Adolescent Mental Health Services (CAMHS) are specialist publicly funded multidisciplinary mental health services that provide assessment, treatment and care coordination for children and adolescents experiencing moderate‐to‐severe mental health difficulties (Robertson and Eapen 2024). Australian studies have identified several limitations affecting the delivery of child and adolescent mental health care, including multidimensional family factors, service fragmentation, long wait times and inadequate clinician training (Paton et al. 2021), emotional burden, increasing service demand, and the challenges in responding effectively to mental health crises (Wintour and Joscelyne 2024). These limitations may be particularly consequential for CYP who require rapid assessment, short‐term intensive intervention, outreach and coordinated transitions between services. However, the current evidence has paid less attention to how these challenges affect the implementation of rapid, mobile crisis response models, particularly in community settings.

In response to these challenges, the NSW Government introduced the Safeguards Team Program (STP), a rapid‐response outreach crisis intervention service for CYP experiencing acute mental health crises, to improve access to timely crisis assessment, intensive short‐term intervention, coordinated referral pathways and continuity of care through warm handovers (i.e., coordinated transfers of care involving direct communication between services) (Eapen et al. 2023). Existing evaluations identified several outcomes of the Macarthur STP, including improved mental health (e.g., reduced stress, anxiety), trauma‐related diagnosis, reduced clinical symptoms, functional improvement and reduced ED use among 201 CYP (Huda et al. 2024, 2026), with limited focus on clinicians' perspectives and experiences of implementing the STP. The current study aimed to explore clinicians' perspectives and experiences of implementing a rapid, mobile mental health crisis response service, the STP for CYP, including perceived barriers, enablers and suggestions for improving mental health services in community mental health settings in the Western Sydney area. Understanding clinicians' perspectives is important because successful implementation depends on workforce engagement, service acceptability, and the identification of operational barriers and facilitators that may influence service sustainability and scalability (Elizalde et al. 2024). Findings from this study may inform the refinement of acute mental health crisis services and support future implementation of community‐based crisis response models for children, young people and their parents/carers/families.

**Figure 1:**
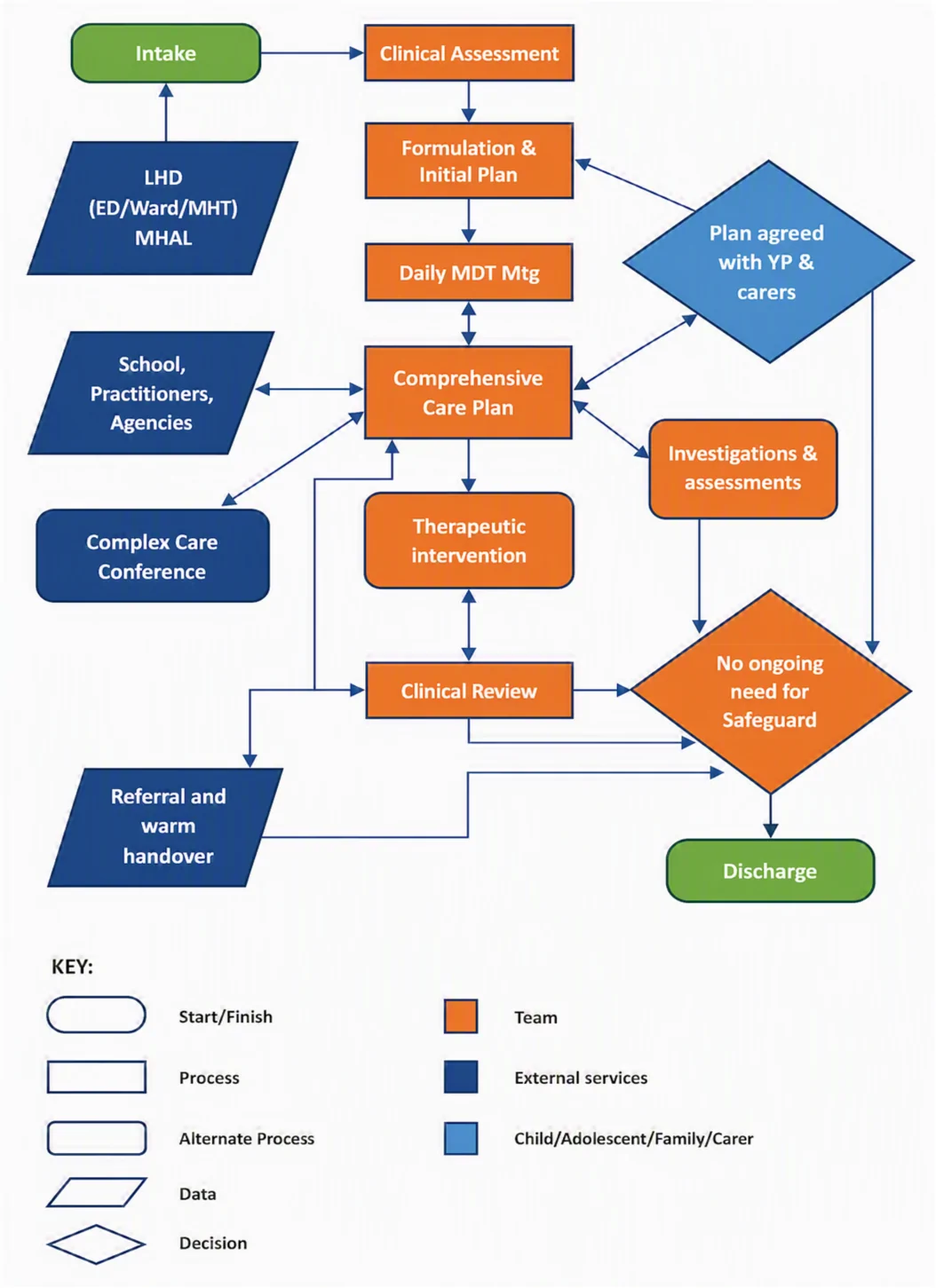
A flow diagram of the processes associated with the Safeguards Team Program (Eapen et al. 2023). CAMHS = Child and Adolescent Mental Health Services, CYP = Children and Young People, ED = Emergency Department, LHD = Local Health District, MDT = Multidisciplinary Team, MDT Mtg = Multidisciplinary Team Meeting, MHAL = Mental Health Access Line, MHT = Mental Health Team, STP = Safeguards Team Programme, YP = Young People. [Color figure can be viewed at wileyonlinelibrary.com]

Figure 1 provides an overview of the STP, including referral pathways, multidisciplinary care processes, therapeutic interventions, service navigation activities, referral and warm‐handover arrangements, and discharge pathways. The STP was developed to provide rapid, intensive support to CYP experiencing acute mental health difficulties. Intervention is typically provided over a 6–8 week period. The STP consists of a multidisciplinary team (MDT) to provide community‐based rapid‐response, recovery‐focused, trauma‐informed comprehensive assessment and brief clinical and psychosocial intervention for 0–17‐year‐olds presenting in mental health crisis (Huda et al. 2026). STP clinicians are experienced mental health professionals who receive specialised training in evidence‐based interventions, including dialectical behaviour therapy (DBT), trauma‐focused cognitive behavioural therapy (TF‐CBT), trauma‐informed care, family‐focused approaches (e.g., Tuning in to Kids, Tuning in to Teens and Confident Carers Cooperative Kids) and culturally responsive practice to support delivery of short‐term crisis interventions. In addition, clinicians participate in multidisciplinary and interagency forums, including Youth Action Meetings, which bring together representatives from health, education, police, child protection and community organisations to coordinate care and support vulnerable young people. As an enhanced acute‐care component within CAMHS, the programme is intended to improve responsiveness to mental health crises, support navigation of complex service systems and strengthen continuity of care through coordinated pathways and facilitated transitions between services (Eapen et al. 2023). Within the Australian mental health service system, CAMHS generally provides ongoing specialist assessment and treatment for children and adolescents (Robertson and Eapen 2024), whereas the STP functions as a rapid‐response outreach service embedded within the broader CAMHS framework, delivering short‐term crisis intervention and service navigation (Eapen et al. 2023). What differentiates the STP from existing service models is its emphasis on proactive outreach, rapid response, short‐term intensive intervention and coordinated service navigation, allowing support to be delivered in settings, including homes, schools, community locations and hospitals, that are most accessible to children, young people and their families (Eapen et al. 2023).

## 2 Materials and Methods

This study used a qualitative design to address the exploratory research question and capture participants' narratives. In‐depth, semi‐structured interviews were employed to elicit detailed accounts of clinicians' perspectives and experiences in implementing the STP, while allowing participants to direct the discussion to relevant topics. The first author piloted the interview questions with two clinicians and revised them to ensure alignment with contextual nuances in language, clinical practice and service delivery (see Supporting Information S1: File 1). This study was conducted and reported in accordance with the Critical Appraisal Skills Program (CASP) Qualitative Checklist.

### 2.1 Participants and Recruitment

This qualitative implementation evaluation study follows the published study protocol (Eapen et al. 2023). Using a purposive sampling approach, all 14 STP clinicians in a metropolitan region of an NSW health service in Australia were invited to participate in this qualitative study. In collaboration with the clinical team leader, the first author presented the study at several (weekly) MDT meetings and invited eligible clinicians to participate. A formal email invitation, study information sheet and consent form were subsequently distributed.

The research team was independent of the clinical Safeguards Team and did not provide clinical interventions to children, adolescents or families receiving care through the programme. Researchers were responsible for participant recruitment, data collection and analysis. This separation between researchers and service providers helped minimise potential role‐related bias and facilitated participants' ability to share their perspectives openly. Finally, nine eligible clinicians consented to participate.

### 2.2 Data Collection

All nine STP clinicians consented to participate, and one research member collected general demographic characteristics of the interview participants. Interviews were conducted between 20 July and 29 September 2023. A mutually convenient time was arranged for a one‐on‐one interview (average time of 45 min) via Microsoft Teams.

### 2.3 Data Analysis

All interviews were audio‐recorded, transcribed by professional transcription services and thematically analysed using an inductive interpretative approach (Braun and Clarke 2006). Three researchers (T.W., T.N. and W.T.W.) coded all interview transcripts using NVivo (version 12). To enhance analytical rigour, a subset of transcripts (*n* = 4) was independently coded by another researcher (M.N.H.). Coding frameworks and emerging themes were compared and discussed within the research team, and any coding discrepancies were resolved through consensus. Several strategies were employed to minimise potential bias and enhance the trustworthiness of the findings. These included the involvement of multiple coders/research team members, independent coding of a subset of transcripts, consensus‐based discussion of coding discrepancies and ongoing reflexive discussions among the research team regarding interpretation of the data. This enabled the identification of common themes and subthemes in clinicians' experiences and perceptions regarding the feasibility, acceptability, appropriateness and sustainability of implementing the STP. Reflexive thematic analysis via inductive coding (Braun and Clarke 2006) allowed data to be organised to explore connections between data elements to develop conceptual items. Once coded, data segments were formally linked to allow themes to emerge and determine the relationships between themes/sub‐themes. The study has been reported in line with the Standards for Reporting Qualitative Research (O'Brien et al. 2014).

### 2.4 Ethical Statement

The study conforms to the principles outlined in the Declaration of Helsinki. (World Medical Association 2013). All methods were carried out in accordance with relevant guidelines and regulations of The National Statement on Ethical Conduct in Human Research. The South Western Sydney Local Health District Human Research Ethics Committee approved this study (Reference Number: 2022/ETH00808), and written informed consent was obtained from all participants before data collection.

## 3 Results

### 3.1 Clinicians' Characteristics

The nine participating STP clinicians comprised one psychiatrist, five mental health nurses and three mental health social workers; one participant was male and eight were female. Six reported having more than 3 years of experience, and three had 1–3 years of experience in mental health.

### 3.2 Main Findings

**Figure 2:**
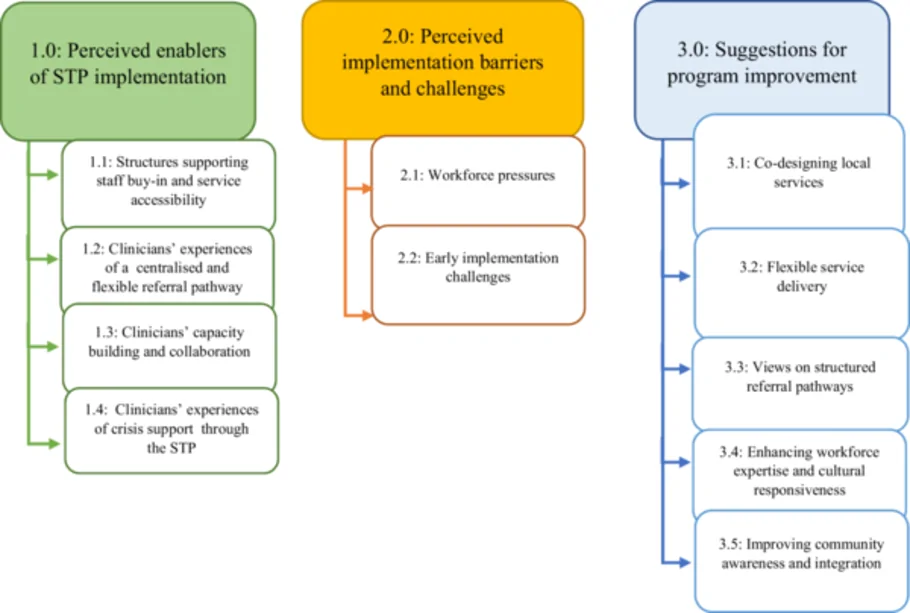
Themes and subthemes identified on perceived implementation enablers, barriers and suggestions for improvement of the Safeguards Team program. [Color figure can be viewed at wileyonlinelibrary.com]

The analysis yielded three overarching themes and 11 subthemes. Figure 2 illustrates these identified themes and subthemes.

#### 3.2.1 Theme 1.0: Perceived Enablers of STP Implementation

##### 3.2.1.1 Subtheme 1.1: Safety Structures Supporting Outreach and Service Accessibility

Most clinicians reported that safety procedures, their understanding of the MoC and perceived that early accessibility and assertive outreach services were important enablers of the STP implementation. Several clinicians provided positive feedback regarding the STP's assertive outreach approach, highlighting its role in improving accessibility for CYP experiencing mental health crises. Participants valued the opportunity to provide rapid intervention in community settings, enabling care to be delivered in locations that were comfortable and accessible for CYP and their families.

I really, really like the assertive outreach that we're able to do, and I think that's where it really differs from other programs, is where we're meeting children and families in their space where they're comfortable.

(P03)

Clinicians also valued the significance of having the STP as an early intervention solution to pick‐up CYP experiencing a mental health crisis early within proximity to their residence/home:

So I really like the fact that we can see that we are at the early intervention is an extremely positive for the community.

(P04)

Clinicians also discussed the principles and supports put in place to help clinicians feel safe when engaging with clients, as well as the necessary structures that enable them to carry out their duties safely. Participants highlighted the importance of a communication system to track clinicians' whereabouts, a system for rapidly following up with a clinician who fails to check in, and the use of a home visit checklist along with other safety measures essential for assertive outreach work with high‐risk clients and families. These processes not only enhance clinicians' safety but also support community‐based outreach and improve accessibility of the STP for CYP and their families, serving as key enablers for the implementation of the STP:

There's all these other processes and procedures that were put in place so that we can feel safe to go out and see patients [CYP] on our own and all of the check‐in processes that are in place now.

(P02)

##### 3.2.1.2 Subtheme 1.2: Clinicians' Experiences of a Centralised and Flexible Referral Pathway

Most clinicians indicated that the introduction of a centralised referral pathway streamlined intake processes, reduced confusion and ensured that referrals to the STP were triaged appropriately. The referral system allowed for a timely response—within 24–48 h—to CYP in need, which was widely supported by participants. This process minimised unnecessary transitions, which clinicians perceived as reducing disruptions to care and supporting continuity of service provision:

So there's lots of sources, but technically all the referrals go through the same pathway, which is through mental health access line.

(P01)

That person was seen within 48 hours and I thought that was a big tick on tick.

(P03)

The flexibility of the STP's extended hours played a crucial role in improving engagement. The service operated 7 days a week, offering late weekday and weekend sessions to accommodate young people and families. While weekday evening in‐reach beyond 7:00 PM was found to interfere with family routines, weekend availability was well received. Parents, particularly those who worked full‐time, appreciated the opportunity to participate more actively in assessments and therapy sessions: ‘I feel like they get to us when they need us’ (P02).

##### 3.2.1.3 Subtheme 1.3: Clinicians' Capacity Building and Collaboration

Providing opportunities to improve clinicians' skills and collaboration through team capacity‐building was an important component that supported the implementation of the programme. Participants affirmed that STP provided better MDT collaboration and integration between services, continuity and after‐care with ‘warm’ handover.

I now have access to community‐based clinicians who have depths of experience and knowledge and my capacity to engage with them on a regular basis on therapies, supervision and engagement increased substantially.

(P08)

All participants alluded to the benefits of opportunities for further training and access to relevant STP courses. They found the training and courses related to the STP were unique and rewarding, helping build capacity and implement what they have learned in a clinical context:

…the first say 6 to 9 months where this wonderful honeymoon period of being able to do a lot of training, engaging with consumers and having a small caseload at the time and just really it was a lot of learning.

(P02)

Participants also talked about the benefits of having a risk huddle daily where broader issues were discussed, allowing STP clinicians opportunities for effective communication, mutual learning and collaboration: ‘Not only the STP clients that are at risk but also any other communications because it's a really good time to capture the entire team before everybody breaks away and attends their appointment’ (P07).

One clinician highlighted that stakeholders recognised the Safeguards team's strong engagement in youth action meetings as particularly proactive and effective in supporting young people:

The other meeting, which is really significant to Safeguards and CAMHS, is the youth action meeting which is held at the police station which we attend monthly as usually a CAMHS or Safeguards client is discussed, so they usually have a list of all the stakeholders across the area that say that Safeguards is the most proactive in terms of involvement.

(P07)

##### 3.2.1.4 Subtheme 1.4: Clinicians Buy‐In and Perceived Values of the STP

Clinicians described strong buy‐in to the STP, driven by their perception that the programme filled an important gap in crisis care for CYP. Working within the STP enabled them to support CYP presenting in crisis who may not meet the intake criteria for other services, which they perceived as a rewarding aspect of their clinical role. Participants emphasised that the STP's capacity to contain crises, equip consumers with coping skills and facilitate referrals to longer‐term services contributed to their sense that the model was valuable and worth implementing. Participants described the STP as addressing an important service gap by providing timely, short‐term, intensive support for young people who might otherwise experience difficulties accessing appropriate crisis care. Clinicians also perceived that the STP helped bridge a critical gap by providing short‐term, intensive specialised support for individuals who might otherwise fall through the cracks. Clinicians viewed the programme as helping contain crises, equip consumers with coping skills and facilitate referrals to longer‐term services, which they viewed as significant benefits of the STP model. This perceived value and alignment with unmet need reinforced clinicians' commitment to the STP:

They [CYP) didn't quite meet the criteria for regular services (CAMHS or CoMHET [Community Mental Health Emergency Team]) and they are kind of the people at times who are likely to fall through the cracks and the six weeks has been enough to be able to contain the crisis, to be able to equip and give them skills and refer them on to a lower intensity, longer‐term service.

(P08)

It definitely services and [provides] need [for] a high proportion of patients [CYP] who wouldn't otherwise fit into a model who are now receiving care and we're being very thoughtful about that care.

(P09)

Clinicians also emphasised the need for a dedicated MoC, such as STP, to ensure that the specificity and acuity of a patient's needs could be appropriately matched. They highlighted that traditional models, such as CAMHS, may not provide the same level of intensive assessment and crisis follow‐up, which reinforced their view that the STP was a necessary and valuable addition to the service system and strengthened their buy‐in to the model:

If you were seeing them under a CAMHS model that would have different expectations; and you might not have the resources to have as intensive an assessment period or kind of follow‐up; Like you couldn't service that acuity or provide that level of diagnostic assessment or opinion back to primary healthcare.

(P09)

Additionally, one participant perceived that the MoC addressed a service gap for young people who otherwise may experience long waiting times or limited access to specialised CAMHS services during periods of crisis, before being referred back to primary care services, such as general practitioners. This perception of filling an unmet need further reinforced clinicians' support for and commitment to the programme:

There's the young people who we refer back to primary care after having given an opinion (in the time of their crisis), and I think that this model services that cohort well, they've previously wouldn't likely have gotten access to specialised CAMH care (in a timely manner or at all).

(P09)

#### 3.2.2 Theme 2.0: Perceived Implementation Barriers and Challenges

##### 3.2.2.1 Subtheme 2.1: Workforce Pressures

Most clinicians reported experiencing an increase in workload several months after the programme's implementation. Clinicians stressed their concerns in meeting the heavy caseload with a very high patient‐to‐clinician ratio, especially resulting in a lack of timely access to the limited medical staff. The service was recruiting senior medical staff and, during this period, temporary consultant support was provided from the regular CAMHS team:

Medical staff have high caseloads as they work between Safeguards and ICAMHS [Infant, Child and Adolescent Mental Health Services] means sometimes patients [CYP] are only seen towards the end of their intervention and may be started on medication shortly before being transferred to ICAMHS/or other step‐down services…

(P03)

It can be quite difficult to access like medical support just because of the caseloads of our doctors.

(P03)

Further, due to high demand and complexity, one clinician reported difficulties in aligning schedules for appointments due to high workload:

Lining up three schedules, my own schedule, the consumer schedule and the parent schedule, sometimes it comes quite difficult or in some cases, the school's schedule plus the doctor's or other stakeholder's schedule…so the compromise has been engaging with (all of) them once.

(P02)

Another clinician reported that the limited STP workforce was resulting in the need to obtain resources from other parts of the service:

And has to basically buddy up with another team, which once again pools resources out of under‐resourced other teams to pinch that other clinician to be able to do their job.

(P01)

##### 3.2.2.2 Subtheme 2.2: Early Implementation Challenges

Clinicians reported a lack of awareness of the MoC and referrers' expectations about the programme's suitability for individual clients. As STP was a new programme, stakeholders lacked clarity about their expectations in the early stages of its implementation. For example, one clinician reported that at the beginning:

And being that we were the first team to launch… they hadn't really, clearly defined exactly what they wanted yet.

(P01)

There was also an initial lack of understanding among other health service providers and parents/guardians regarding expectations for the programme in its early stages. Clinicians perceived that uncertainty regarding the purpose and expectations of the STP among referrers and parents/guardians created challenges during the early stages of implementation:

It gets confusing for other health services that are making referrals, but it also gets confusing for the parents.

(P03)

#### 3.2.3 Theme 3.0: Suggestions for Programme Improvement

##### 3.2.3.1 Subtheme 3.1: Co‐Designing Local Services

Clinicians emphasised the importance of incorporating location‐specific, co‐designed referral pathways developed collaboratively with key stakeholders, including clinicians, community organisations, consumers, their families and carers, to contextualise services within their specific environments. They highlighted that tailoring the services to the unique needs and characteristics of the local area is crucial for ensuring the project's relevance and effectiveness. Most clinicians perceived that involving key stakeholders in the design and refinement of local service pathways could improve engagement, responsiveness and implementation of the STP within different communities. A clinician praised the idea of co‐design, reflecting on how the involvement of stakeholders in the design process could foster better outcomes and engagement: ‘I absolutely loved co‐design. You know’ (P08).

##### 3.2.3.2 Subtheme 3.2: Flexible Service Delivery

Clinicians working within the Safeguards Team offered several suggestions for improving the MoC across the service system, particularly emphasising the need for greater flexibility in the programme's frequency and duration rather than fixed timelines, and follow‐ups that align with individual patient needs. A clinician echoed this sentiment, noting that the shorter duration can sometimes make the process feel rushed, leaving CYP without the full benefits of treatment.

… I think it feels rushed sometimes… ideally I'd like to work with them longer and like we've accomplished some good, but we definitely could have done a lot more with the younger [CYP].

(P09)

This also highlights the importance of customising care models within the overall service system with more tailored matching of clients for the Safeguards program and warm handover to other parts of the service for those needing longer duration of care to address CYP's needs and foster more effective and meaningful patient relationships and care.

In terms of patient engagement, a few clinicians felt the pressure to build rapport with parents and consumers:

…having that like just building that trust and relationship with your consumers is like already challenging.

(P02)

Clinicians also suggested that they would like to see lower consumer‐to‐clinician ratios so that they could provide an adequate and meaningful level of engagement:

I do remember that I've noticed the difference when I was holding 10 to 12, my capacity to engage with patients [CYP] was substantially reduced.

(P02)

##### 3.2.3.3 Subtheme 3.3: Views on Structured Referral Pathways

Clinicians emphasised the importance of establishing a clear, structured process for triage, managing referrals and patient care pathways from the outset to ensure that CYP are directed to the most appropriate service from the beginning. This structured approach would reduce inefficiencies, improve coordination and ultimately lead to better outcomes for both CYP and clinicians:

And like, from the get go try and have some sort of assistance, some sort of a structure in terms of like exactly what we've talked about …. what does that referral pathway look like, you know, how do we triage and you know when we allocate, how do we do that, you know initial assessment and you know just like having from get go what that looks like, I think we'll cut out a lot of I don't know like fluffing around.

(P08)

In particular, clarity of the STP referral pathway was raised by one clinician to suit the needs of the programme within individual health services and ultimately across the state: ‘So I think it could probably be redefined in a way that there's a clear distinction between who is a Safeguards referral and who is a regular CAMHS referral’ (P03).

Further, several clinicians recommended establishing a referral system that accepts referrals from multiple sources in addition to the ED, coordinated through a common team leader and intake coordinator, to strengthen triage processes and improve consistency in referral decision‐making.

…So Safeguards used to get direct referrals from CoMHET, but we've realised that we can also receive patients [CYP] from different referral sources if we have a common team leader and one intake person,… we may as well utilise them to be able to make and support make decisions.

(P02)

##### 3.2.3.4 Subtheme 3.4: Enhancing Workforce Expertise and Cultural Responsiveness

Clinicians recognised the importance of ensuring that the Safeguards Team includes clinicians with specific expertise to effectively meet the diverse needs of the population they serve. In particular, participants highlighted the need for specialised knowledge in Aboriginal healthcare and culturally and linguistically diverse (CALD) communities to provide more culturally responsive care for these consumer groups: ‘That's going to pose more challenges as well with the Aboriginal identified’. (P06).

##### 3.2.3.5 Subtheme 3.5: Improving Community Awareness and Integration

Participants highlighted the need to increase community awareness about programmes like the STP to ensure that CYP, carers and potential referrers understand mental health crises and the importance of seeking early support and intervention. For example, participants suggested:

So the understanding and the awareness, you know, the promotion of the Safeguards program is not there and perhaps it would be beneficial if that would be more made aware like and then there's the distinction between those.

(P03)

I feel like service education for the community is so, it's so important.

(P04)

I mean so perhaps greater community awareness of the model would be beneficial.

(P09)

Beyond community education, clinicians also underscored the importance of strong integration between STP and regular CAMHS teams to facilitate seamless transitions for young people post‐discharge. The ability of the Safeguards Team to integrate within the broader CAMHS framework was recognised as a key strength, allowing access to a wider pool of expertise and resources while still maintaining STP's specialised focus:

I think that the modality where the Safeguards team is attached to a CAMHS team really does lend itself to having all of the positives of accessing the pool of support whilst also being niche and different enough to still have meaningful work.

(P02)

## 4 Discussion

Our study findings demonstrated strong endorsement of the STP by the clinicians working on the team, who highlighted valued features and enablers of the programme that have supported its success thus far, as well as some challenges. These identified enablers and advantages contribute to the growing evidence base on mental health service models about effective interventions (Berardi et al. 2024; Buljac‐Samardzic et al. 2020; Fehily et al. 2023; Isaacs and Mitchell 2024; McGorry and Mei 2018). The early and assertive outreach approach during crisis was greatly appreciated by the clinicians, enabling successful implementation. This parallels findings in mental health research suggesting that early intervention can significantly mitigate the escalation of crisis and improve long‐term outcomes (McGorry and Mei 2018). Our findings on clinicians' perceptions of having a centralised and flexible model for timely and accessible care were also an effective feature of the STP, supporting timely and accessible care, comparable to findings in community mental health frameworks where integrated care models have demonstrated improved patient outcomes, streamlined service delivery and enhanced coordination among MDTs to provide holistic and accessible support (Berardi et al. 2024; Fehily et al. 2023; Isaacs and Mitchell 2024). Furthermore, STP's flexibility in operating hours and its investment in clinicians' capacity‐building align with research highlighting that adaptable models and well‐trained teams enhance service effectiveness in high‐demand mental health settings (Buljac‐Samardzic et al. 2020).

A significant strength identified by STP participants was the programme's broad eligibility criteria, ensuring that young people and their families receive the right care at the right time through a warm handover process. This feature minimises disruptions in care and prevents service users from having to navigate multiple agencies independently, so that they do not need to ‘repeat their story’ unnecessarily or go from one agency to another in order to find the right service (National Mental Health Commission 2021). Clinicians emphasised that this approach has paved the way for meeting the needs of the CYP and their families.

Study participants perceived that the STP addressed service gaps, particularly for CYP experiencing a mental health crisis who may not meet the intake criteria for other services. They recognised that the STP played a crucial role in bridging this gap by offering timely short‐term, intensive support to individuals who might otherwise be left without appropriate care. This aligns with existing literature on crisis intervention services, which underscores the importance of timely and accessible care in preventing symptom escalation and reducing emergency department presentations (Harvey et al. 2023; Johnson et al. 2022; O'Brien et al. 2016; Roennfeldt et al. 2021). In our study, the clinicians perceived that the STP helped contain crises, equip consumers with essential coping skills, and facilitate referrals to longer‐term services. These were seen as a key benefit, consistent with findings from community‐based mental health frameworks that emphasise early intervention and transitional support (Harvey et al. 2023).

However, alongside these strengths, our study findings highlight barriers to implementation that mirror broader challenges in mental health service delivery while also presenting unique complexities. A primary barrier identified in this study was clinicians' struggle with the demands of working within a crisis intervention model, indicating a need for more specific training and capacity‐building initiatives—a challenge also reflected in studies on crisis mental health services (Hollander et al. 2012). For example, a study by De Hert (2020) emphasised that high‐stress environments increase the risk of burnout, particularly without adequate crisis training and support, limiting the sustainability of crisis‐focused models. While the STP attempted to address this through capacity‐building, the rapid increase in demand and workload suggests that traditional staffing levels and skills training may be insufficient for high‐demand environments, a finding consistent with literature on mental health workforce challenges (Cleary et al. 2020).

The current study's suggestions for improving the STP align with recommendations from broader mental health services literature, underscoring the need for adaptability, community integration and enhanced referral processes (Elizalde et al. 2024). One significant suggestion was developing a more flexible model for the duration, frequency and engagement of patient–clinician encounters. The STP program's extended hours of operation align with research emphasising that mental health services benefit from operational flexibility, as rigid hours often fail to meet the needs of individuals in crisis, especially in underserved communities (Mongelli et al. 2020). Expanding operational hours and adaptability has shown promise in similar models, enhancing accessibility and reducing emergency department visits for mental health crises (Mao et al. 2023; Vacher et al. 2024). However, while flexibility is advantageous, some studies caution that extended hours can strain workforce capacity without adequate staffing and resources, underscoring the need for thoughtful resourcing when implementing this recommendation.

### 4.1 Implications for Health Practice, Policy and Research

Drawing from these findings, several implications for mental health practice, policy and future research emerge:

- A more flexible service ecosystem is needed, where STP provides rapid access to care followed by warm handovers to internal or external services for continued intervention and support. Geographical proximity should also be considered to ensure accessible after‐care options.
- Initial and ongoing co‐design with clinicians, consumers and carers is critical to refining the STP model in response to process evaluations and evolving community needs.
- Capacity‐building and workforce adaptability must be prioritised, with continuous improvement cycles responding to workforce challenges and changes in community mental health needs.
- Community awareness initiatives should be expanded to improve understanding of mental health crisis services, such as STP and available support pathways, including strengthening access to clear and readily available information about the STP and referral pathways for referrers (e.g., Mental Health Access Lines, support services, general practitioners, schools and community organisations), as well as children, young people, families and carers. This may include providing accessible online information and developing patient and carer information resources outlining the purpose of the STP, eligibility criteria, referral pathways, what the intervention involves and how the service integrates within the broader child and adolescent mental health service system. Such resources could be made available online and provided at the commencement of referral and engagement with the service.
- Recruitment and training of staff with expertise in Aboriginal and CALD communities should be prioritised.

### 4.2 Strengths and Limitations

This study has several strengths, including the use of semi‐structured interviews to gain in‐depth insights from clinicians and the inclusion of all eligible STP clinicians working at the service at the time of data collection. Rigorous interpretive and quality‐control measures were applied, enhancing the trustworthiness of the findings. Furthermore, the study's findings can inform the design and implementation of other novel service development approaches. However, several limitations must be considered. The study was conducted at a single metropolitan site in NSW and involved a limited number of clinicians, so the findings may not be transferable to other sites or to regional or remote areas. Future research should explore perspectives from multiple metropolitan and regional sites and more clinicians from more diverse occupational backgrounds. Moreover, this study sought only clinicians' experiences and views. Future research could include the perspectives of CYP and carers, as well as referrers. Potential biases, including recall bias and selection bias, could have influenced data interpretation. Nonetheless, as an exploratory pilot study, this research provides valuable insights into the feasibility, enablers and barriers of STP's implementation.

## 5 Conclusions

Our findings suggest that clinicians perceived the STP as having the potential to address a gap in the service system for young people requiring rapid outreach specialist mental health crisis response. It is expected that findings on clinicians' perceptions will be crucial to evaluating the impact and implementation outcomes of the new MoC, such as the STP. The findings can also inform the design and implementation of other novel service development approaches. Future research should incorporate co‐design approaches before implementation and quantitative evaluations to assess clinical outcomes and consumer perspectives. Our findings contribute to the growing evidence base on CYP's mental health crisis intervention programmes, providing insights that can inform future acute crisis response service models and policy development.

## Author Contributions

Conceptualisation: Teresa Winata, Weng Tong Wu, and Md Nazmul Huda. Data curation: Teresa Winata, Weng Tong Wu, and Md Nazmul Huda. Formal analysis: Teresa Winata, Weng Tong Wu, Md Nazmul Huda, and Thomas Nguyen. Funding acquisition: Valsamma Eapen and Rajeev Jairam. Investigation: Teresa Winata, Md Nazmul Huda, and Weng Tong Wu. Methodology: Md Nazmul Huda, Teresa Winata, and Thomas Nguyen. Project administration: Teresa Winata, Md Nazmul Huda, and Weng Tong Wu. Resources: Md Nazmul Huda, Teresa Winata, Valsamma Eapen, and Rajeev Jairam. Software: Teresa Winata, Weng Tong Wu, Md Nazmul Huda, and Thomas Nguyen/ Supervision: Valsamma Eapen, Rajeev Jairam, and Michael Bowden. Validation: Md Nazmul Huda, Teresa Winata, Valsamma Eapen, Michael Bowden, and Rajeev Jairam. Writing – original draft: Teresa Winata, Md Nazmul Huda, Weng Tong Wu, and Thomas Nguyen. Writing – review and editing: Valsamma Eapen, Rajeev Jairam, Thomas Nguyen, Md Nazmul Huda, and Michael Bowden.

## Funding

The authors have nothing to report.

## Ethics Statement

The study conforms to the principles outlined in the Declaration of Helsinki (World Medical Association 2013). All methods were carried out in accordance with relevant guidelines and regulations of The National Statement on Ethical Conduct in Human Research. The South Western Sydney Local Health District Human Research Ethics Committee approved this study (Reference Number: 2022/ETH00808), and written informed consent was obtained from all participants before data collection.

## Conflicts of Interest

The authors declare no conflicts of interest.

## AI Disclosure Statement

No generative artificial intelligence (AI) tools were used in the writing, editing, data analysis, or preparation of this manuscript. All content was developed solely by the authors.

## Supporting information


Supporting File

## Data Availability

The data that support the findings of this study are available on request from the corresponding author. The data are not publicly available due to privacy or ethical restrictions.
