# Supplementary material for: Clinicians' Perspectives and Experiences of Barriers, Enablers and Implementation of a Child and Adolescent Mental Health Crisis Service: A Qualitative Evaluation of the Safeguards Team Program
Source: J Child Adolesc Psychiatr Nurs. 2026 Sep 9;39(4):e70075. doi: 10.1111/jcap.70075 (PMC13555513; doi:10.1111/jcap.70075)
Supplement: Supplementary file 1 [file JCAP-39-e70075-s001.docx]

**Supplementary File 1.** Interview Guide Questions for Participants

1. How has the implementation of the Safeguards Team program proceeded in this LHD? How did it begin and how did it proceed, describe the process, what is the current situation?

- How did the change in the work process take place in practice?

2. Describe your experience in providing, conducting, coordinating or supporting a patient-centred outreach and follow-up for people experiencing a mental health crisis in this LHD.

- Can you give me an example or some examples of these experiences and tell me how that work out?
- When do you usually provide these services or do these work?
- Is this something you do routinely? Or not?
- What are some of the tools you use?
- How do you work with other health workers/professionals or members within your service organisation?
- How do you see your role impacting the people/consumers you see on a regular basis?
- What factors have you found useful when providing this support?
- What factors do you find challenging when providing this support?

3. Did the COVID-19 pandemic and lockdowns impact how you work with your clients/consumers? Did you have to adopt to telehealth consultations/appointments?
4. Describe your experiences of the referral system/pathway for the Safeguards Teams program. Do you have any feedback about the referral guidelines/information provided?

*NOTE: Referrals into the Safeguards Team program occur via the children and adolescent mental health service. Further, anyone can call the mental health emergency line and request a Safeguards Teams referral for themselves or others. A Safeguards Team staff member will call and arrange to meet consumers in the community or at their home where appropriate. In addition to providing compassionate acute care by a Safeguards team member, the clients are supported for the next steps before being discharged.*

5. What kind of training or support have you received being a Safeguards clinician or team member role in this LHD (from your supervisor, in your workplace, or in general)?

- What kind of orientation/support would you like to receive?
- Have you personally experienced that you have received adequate information/training about the service?
- Have you received information on where to get help with service when problems occur?

6. Would you recommend the implementation of Mental Health Clinician approach to be embedded as part of children and adolescent mental health mental services and model of care in this LHD? Why/Why not?
7. Are there any additional/missing components that you could recommend for future implementation for such program like the Safeguards Teams program?
8. Is there anything I have not asked about that you would like to share about your experience as a mental health clinician or Safeguards team member in this program?
